# Effects of acupuncture on cartilage p38MAPK and mitochondrial pathways in animal model of knee osteoarthritis: A systematic evaluation and meta-analysis

**DOI:** 10.3389/fnins.2022.1098311

**Published:** 2023-01-11

**Authors:** Jiang-nan Ye, Cheng-guo Su, Yu-qing Jiang, Yan Zhou, Wen-xi Sun, Xiao-xia Zheng, Jin-tao Miao, Xiang-yue Li, Jun Zhu

**Affiliations:** ^1^School of Acupuncture–Moxibustion and Tuina, Chengdu University of Traditional Chinese Medicine, Chengdu, China; ^2^Xiyuan Hospital, China Academy of Chinese Medical Sciences, Beijing, China; ^3^Graduate School, Guangzhou University of Chinese Medicine, Guangzhou, China

**Keywords:** knee osteoarthritis, acupuncture, animal models, p38MAPK pathways, mitochondrial pathways, cytokines, meta-analysis

## Abstract

**Background:**

Most previous studies on acupuncture in the treatment of knee osteoarthritis (KOA) have focused on improving functional efficacy and safety, while related mechanisms have not been systematically reviewed. Acupuncture modulates cytokines to attenuate cartilage extracellular matrix degradation and apoptosis, key to the pathogenesis of KOA, but the mechanisms are complex.

**Objectives:**

The purpose of this study is to assess the efficacy of acupuncture quantitatively and summarily in animal studies of KOA.

**Methods:**

Nine databases including PubMed, Embase, Web of Science (including Medline), Cochrane library, Scopus, CNKI, Wan Fang, and VIP were searched to retrieve animal studies on acupuncture interventions in KOA published since the inception of the journal. Relevant literature was screened, and information extracted. Meta-analysis was performed using Revman 5.4 and Stata 17.0 software.

**Results:**

The 35 included studies involved 247 animals, half of which were in acupuncture groups and half in model groups. The mean quality level was 6.7, indicating moderate quality. Meta-analysis showed that acupuncture had the following significant effects on cytokine levels in p38MAPK and mitochondrial pathways: (1) p38MAPK pathway: It significantly inhibits p38MAPK, interleukin-1beta (IL-1β), tumor necrosis factor alpha (TNF-α), phosphorylated (p)-p38MAPK, matrix metalloproteinase-13 (MMP-13), MMP-1, a disintegrin and metalloproteinase with thrombospondin motifs-5 (ADAMST-5) expression, and significantly increased the expression of collagen II and aggrecan. (2) mitochondrial pathway: It significantly inhibited the expression of Bcl-2-associated X protein (Bax), cysteine protease-3 (caspase-3), caspase-9, and Cytochrome-c (Cyt-c). And significantly increased the expression of B cell lymphocytoma-2 (Bcl-2). In addition, acupuncture significantly reduced chondrocyte apoptosis, Mankin’s score (a measure of cartilage damage), and improved cartilage morphometric characteristics.

**Conclusion:**

Acupuncture may inhibit cytokine expression in the p38MAPK pathway to attenuate cartilage extracellular matrix degradation, regulate cytokines in the mitochondrial pathway to inhibit chondrocyte apoptosis, and improve cartilage tissue-related phenotypes to delay cartilage degeneration. These findings provide possible explanations for the therapeutic mechanisms and clinical benefits of acupuncture for KOA.

**Systematic review registration:**

https://inplasy.com, identifier INPLASY20 2290125.

## 1. Introduction

Knee osteoarthritis (KOA) has become a socially prevalent and disabling disease, with severe pain and impaired function seriously affecting quality of life and imposing a significant economic burden on many developed countries (Bedenbaugh et al., 2021). The pathology of KOA is characterized by apoptosis of chondrocytes and progressive destruction of articular cartilage, restoring the integrity and function of articular cartilage plays a key role in preventing or delaying the progression of KOA (Goldring and Berenbaum, 2015). However, the lack of nerves, blood or lymphatic vessels in articular cartilage limits the scope for repair after injury (Thomas et al., 2021). Current treatments in the field of KOA cartilage repair are complex, cause significant autologous damage, and may only reach the periosteal rather than cartilage level (Pallante et al., 2009; Hunziker et al., 2015), therefore other non-pharmacological therapies with alternative and preventive effects and fewer side effects are needed.

Acupuncture, a kind of traditional Chinese non-pharmaceutical treatment, has therapeutic and preventive effects on KOA (Corbett et al., 2013). Acupuncture was suggested for the treatment of KOA as early as 2019 by the International Guidelines for the Non-Surgical Management of Osteoarthritis (OA) by the OA Research Society (Bannuru et al., 2019) and the American College of Rheumatology (ACR)/Arthritis Foundation(AF) management guidelines (Kolasinski et al., 2020). In the acute phase of KOA, acupuncture offers quick alleviation of pain and dysfunction, according to two randomized controlled trials published in *Pain* and *Arthritis & Rheumatology* (Lin et al., 2020; Tu et al., 2021). This sensational finding provides a powerful response to a study (Hinman et al., 2014) published in *JAMA* that concluded that acupuncture is ineffective in the treatment of arthritis, and has important implications for the future of acupuncture clinical practice internationally, as well as for the inclusion of acupuncture in mainstream medical clinical treatment guidelines and health insurance. At the same time, a growing body of evidence (Jie et al., 2021; Qin et al., 2022) suggests that acupuncture can repair cartilage microarchitecture and delay or even reverse cartilage defects by altering KOA phenotypic changes caused by the inflammatory environment, slowing cartilage matrix degradation, and inhibiting chondrocyte apoptosis.

The therapeutic mechanism of acupuncture is complex and in KOA may involve modulating the relevant pathway signals in cartilage to inhibit disease progression. In KOA, inflammatory factors mediate chondrocyte differentiation by signaling to various transcription factors through intracellular signaling pathways. These induce a change from chondrocyte to fibroblast phenotype, prompting chondrocyte apoptosis n the joint and fibrosis of the surrounding tissue (Xie et al., 2021). The pathways include mitogen-activated protein kinase (MAPK) and mitochondrial signaling pathways (Brown et al., 2008; Sun et al., 2019). MAPK plays an important role in regulating various cellular processes such as apoptosis, survival, proliferation, and migration. For example, isoform p38MAPK has been widely used as a target to inhibit cytokines for the treatment of inflammatory diseases (Schindler et al., 2007). p38MAPK signaling (Zhou et al., 2015) is essential for the expression and activity of MMP and ADAMTS, which are protein hydrolases that contribute to matrix degradation and cartilage destruction. In the mitochondrial pathway, B cell lymphocytoma-2 (Bcl-2) and Bcl-2-associated X protein (Bax) are the main factors regulating apoptosis, and apoptosis-associated protein cysteine protease 3 (Caspase-3) is the final apoptosis execution molecule (Zhang et al., 2014).

Acupuncture can delay KOA progression by modulating p38MAPK and the mitochondrial signaling pathway (Liao et al., 2016; Liu J. W. et al., 2021), but the mechanism by which it effects cartilage repair in KOA has not been systematically studied. Systematic review methods facilitate evidence-based clinical decision making (Ritskes-Hoitinga et al., 2014), indicate gaps in research, reduce unnecessary duplication of studies, and support the “replacement, refinement, and reduction of animals” principle in animal research (Murphy and Murphy, 2010). Therefore, the purpose of this review is to systematically review the research on the effect of acupuncture on cartilage repair signaling pathways in animal models of KOA, to quantitatively assess pooled effects, and to provide indicators for future clinical studies.

## 2. Materials and methods

### 2.1. Search strategy

In conducting this systematic review, we adhered to the Preferred Reporting Items for Systematic Reviews and Meta-Analyses (PRISMA) guidelines (Page et al., 2021). The review was conducted in accordance with our previously published protocol (INPLASY202290125).^1^ Two authors (Ye, JN and Su, CG) independently searched the databases of Pubmed, Embase, Web of Science (including Medline), Cochrane library, Scopus, CNKI, Wan Fang, and VIP. No date constraints were imposed on the search, which was completed in September 2022. Take PubMed for example, the specific search formula is: (*Acupuncture* [Title/Abstract] OR *Osteoarthritis, Knee* [Title/Abstract] OR *Models, Animal* [Title/Abstract]). Each search term was used individually or in combination, and the specific search strategy is described in Supplementary material. In addition, the reference lists of studies included in the review were manually screened for further studies.

### 2.2. Eligibility criteria

For this study, we devised the following inclusion and exclusion criteria in full line with the PICOS (Participation, Intervention, Comparison, Outcome, Type of Study) principles:

Participant type (P): all research on animals with KOA were considered, regardless of species, gender, month of age, or modeling approach, but the indicator measured must be derived from cartilage tissue. Non-animal and non-chondrogenic level KOA studies were omitted.

Intervention type (I): Acupuncture was used to treat KOA in the intervention group, with no limits on method, time per treatment, treatment course, or acupuncture locations. If several electro-acupuncture frequencies or intervention sessions were available, to better fit the characteristics of the function of acupuncture, the highest frequency or longest intervention duration was chosen for analysis. Excluded were studies in which non-acupuncture or acupuncture was not the principal intervention.

Comparison type (C): The model group was modeled only without any interventions.

Outcome type (O): (1) Main result markers in cartilage tissue: cytokines of the p38MAPK pathways (including p38MAPK, IL-1β, TNF-α, p-p38MAPK, MMP-13, MMP-1, ADAMST-5, collagen II, aggrecan) and mitochondrial pathways (including Bax mRNA, Bcl-2 mRNA, Caspase-3 mRNA, Caspase-9 mRNA, Bax, Bcl-2, caspase-3, Caspase-9, Cyt-c); (2) secondary outcome indicators: chondrocyte apoptosis rate, Mankin’s score measuring the extent of cartilage damage and cartilage morphometric score, including the cartilage and subchondral volume ratio/total volume ratio (BV/TV), average thickness of trabecular column structures (Tb.Th), average number of trabeculae per unit length (Tb.N), and average distance between trabeculae (column structures) (Tb.Sp).

Study type (s): All randomized controlled trials that looked at the cartilage component of acupuncture interventions in KOA animal models were considered. There were no clinical case reports, reviews, or conferences. No language limits were applied to ensure that the most extensive research could be provided.

### 2.3. Data extraction

Two authors independently extracted data from articles meeting the inclusion criteria. The following were extracted: first author’s name and year of publication, animal species, animal sex and number of animals in each group, modeling methods, interventions, duration of treatment, outcome indicators and sample tissue. When primary data were missing from the included literature or were only presented graphically, attempts were made to contact the authors to obtain the original data. If the authors did not respond, the values in the graphs were extracted using GetData Graph Digitizer 2.26 software (Wang R. et al., 2021).

### 2.4. Quality assessment

Two researchers assessed the methodological quality of each study using a 10-item checklist modified from the Collaborative Methods for the Analysis and Review of Experimental Research Animal Data (CAMARADES) checklist (Lee and Kim, 2022). These included: sample size calculations, statements describing temperature and humidity control, randomization of groups, use of reasonable KOA models, assessment of modeling success, use of anesthetics with no apparent specificity, blinding of results, compliance with animal ethics regulations, published in a peer-reviewed journal or have passed peer review, and declaration of potential conflicts of interest. A sum of quality scores for each article was calculated out of a maximum score of 10. Disagreements between the researchers were resolved by discussion with a third author.

### 2.5. Statistical analysis

Meta-analysis was performed using Cochrane Collaboration Network RevMan 5.4 and Stata 17.0 software. The data of this study were continuous variables. Standardized mean difference (SMD) was used as an indicator of effect size when the units of measurement information differed between studies, when the values differed significantly, or when the methods of measurement differed, and mean difference (MD) was used in the remaining cases. All effect sizes were expressed as 95% confidence intervals (95% CI). When the heterogeneity among included studies was low (*P* > 0.1 and *I*^2^ ≤ 50%), the fixed-effects model (FEM) was used for analysis; when *P* ≤ 0.1 or *I*^2^ > 50%, heterogeneity was deemed to be present, and subgroup analysis and sensitivity analysis were used to investigate the sources of heterogeneity. The reasons for heterogeneity were analyzed to determine whether Meta-analysis could be performed using random effects models (REM), but if there was significant heterogeneity between studies, only descriptive qualitative analysis was performed without combining it. Sensitivity analysis was performed by using the single-study method of Revman removal to remove “high risk of bias” literature one by one and sensitivity plotting with Stata to assess the reliability and stability of positive study results. We investigated whether potential confounding factors could influence the acupuncture effects of cytokines like MMP-13, which were highly heterogeneous in the study results. We also carried out additional analyses stratified by variables that are frequently disregarded but crucial in animal experiments, such as animal population selection and animal sex configuration. For differences in estimates between these subgroups, *P* < 0.05 were deemed significant. Potential publication bias was assessed by visualizing asymmetries in funnel plots (≥10 studies) by combining Egger’s test and Begg’s test.

## 3. Results

### 3.1. Literature screening results

**FIGURE 1:**
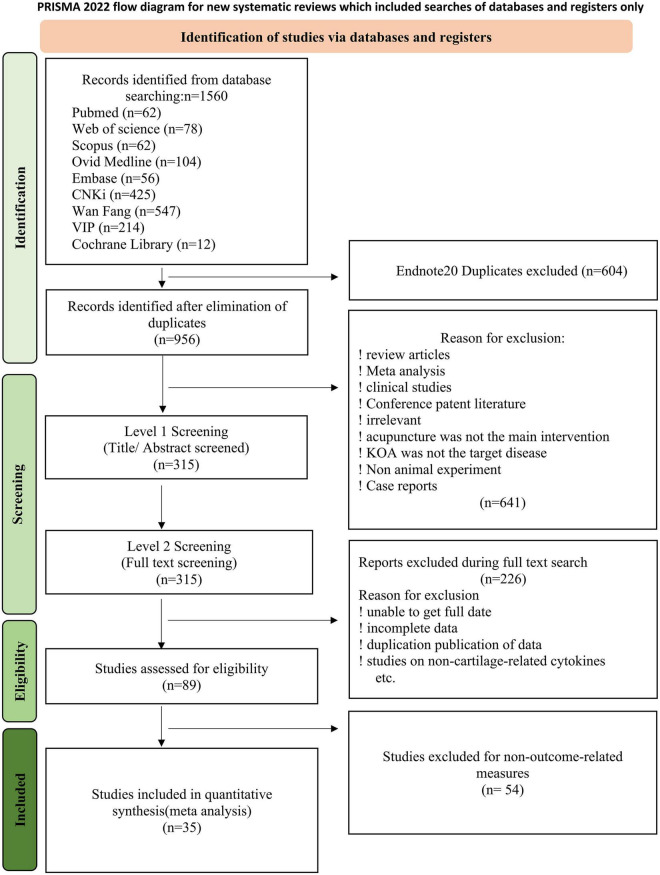
Flow diagram of study selection process.

Literature search yielded 1,560 articles, of which 1,186 were in Chinese, 372 in English, and two in Korean. Of these, 604 duplicates were excluded using Endnote20 check. After reading the abstracts and titles of the remaining 956 papers a further 632 (including reviews, systematic reviews, conferences, patents, subject irrelevant, acupuncture non-primary therapies, KOA non-target diseases, case reports, and non-animal studies) were excluded based on the predetermined criteria. On accessing the remaining 315 articles, 226 were excluded due to unavailable full text, incomplete data, duplicate data publication, or non-chondrogenic level studies. About 89 that met the basic requirements and 54 articles with irrelevant outcome indicators. Ultimately 35 articles were included in the meta-analysis, 25 of which were in Chinese and 10 in English. Figure 1 shows details of the literature search process.

### 3.2. Quality of the literature

The included studies’ quality scores ranged from 4 to 9 out of 10: one study (Zhou et al., 2018) received a score of 9, eight studies (Liao et al., 2016; Ma et al., 2017; Wu et al., 2019, 2022; Wang et al., 2020; Jie et al., 2021; Liu J. W. et al., 2021; Zhu S. Q. et al., 2021) received a score of 8, 16 studies (Xiong et al., 2012; Liang et al., 2015; Fu et al., 2016a; Fu et al., 2016b; Xi et al., 2016; Yu, 2016; Liu N. G. et al., 2018; Wan et al., 2019; Zhang et al., 2019a; Zhang et al., 2019b; Wang, 2020; Chen, 2021; Liu H. et al., 2021; Zeng et al., 2021; Zhu D. Y. et al., 2021; Qin et al., 2022) received a score of 7, four studies (Liang, 2015; Lin and Xu, 2019; Chen et al., 2020; Liu D. et al., 2021) received a score of 6, three studies (Wu et al., 2018; Liu, 2019; Wan et al., 2021) received a score of 5, and three studies (Bao et al., 2011; Liu D. et al., 2017; Wang D. et al., 2021) received a score of 4. There was no research that reported on the sample size calculation. Except for five investigations (Bao et al., 2011; Liu D. et al., 2017; Wu et al., 2018; Wan et al., 2021; Wang D. et al., 2021), all detailed laboratory temperature and humidity control. All research showed randomization of animal groups and employed adequate animal models, and all but three studies (Xiong et al., 2012; Liao et al., 2016; Liu H. et al., 2021) assessed modeling success and failure metrics. Thirty-three research employed anesthetic supplies that had no effect on the experiment, while the remaining six experiments (Bao et al., 2011; Liu D. et al., 2017; Wu et al., 2018; Liu, 2019; Liu J. W. et al., 2021; Wang D. et al., 2021) did not require anesthesia during the experiment. Three studies (Liao et al., 2016; Zhou et al., 2018; Liu J. W. et al., 2021) used blinding during the statistical analysis of the data. Nine research (Bao et al., 2011; Liang, 2015; Liu D. et al., 2017; Lin and Xu, 2019; Liu, 2019; Chen et al., 2020; Liu D. et al., 2021; Wan et al., 2021; Wang D. et al., 2021) did not declare compliance with animal welfare regulations and a further 11 (Xiong et al., 2012; Liao et al., 2016; Ma et al., 2017; Zhou et al., 2018; Wu et al., 2019; Wang et al., 2020; Jie et al., 2021; Liu H. et al., 2021; Liu J. W. et al., 2021; Zhu S. Q. et al., 2021; Wu et al., 2022) reported no possible conflict of interest. Thirty-two studies were published in peer-reviewed journals, and five (Liang, 2015; Yu, 2016; Liu, 2019; Wang, 2020; Chen, 2021) were master’s or doctorate theses that were peer-reviewed at the time of defense and so met the standards as well. For more information, see Table 1.

**TABLE 1:** Quality assessment.

References	Q1	Q2	Q3	Q4	Q5	Q6	Q7	Q8	Q9	Q10	Total
Chen et al., 2020		✓	✓	✓	✓	✓			✓		6
Fu et al., 2016b		✓	✓	✓	✓	✓		✓	✓		7
Liao et al., 2016		✓	✓	✓		✓	✓	✓	✓	✓	8
Zhu D. Y. et al., 2021		✓	✓	✓	✓	✓		✓	✓		7
Wang, 2020		✓	✓	✓	✓	✓		✓	✓		7
Chen, 2021		✓	✓	✓	✓	✓		✓	✓		7
Liu D. et al., 2021		✓	✓	✓	✓	✓			✓		6
Wu et al., 2019		✓	✓	✓	✓	✓		✓	✓	✓	8
Wang et al., 2020		✓	✓	✓	✓	✓		✓	✓	✓	8
Jie et al., 2021		✓	✓	✓	✓	✓		✓	✓	✓	8
Lin and Xu, 2019		✓	✓	✓	✓	✓			✓		6
Liu D. et al., 2017			✓	✓	✓				✓		4
Wu et al., 2018			✓	✓	✓			✓	✓		5
Xi et al., 2016		✓	✓	✓	✓	✓		✓	✓		7
Zhang et al., 2019a		✓	✓	✓	✓	✓		✓	✓		7
Bao et al., 2011			✓	✓	✓				✓		4
Liu H. et al., 2021		✓	✓	✓		✓		✓	✓	✓	7
Liang, 2015		✓	✓	✓	✓	✓			✓		6
Yu, 2016		✓	✓	✓	✓	✓		✓	✓		7
Liu N. G. et al., 2018		✓	✓	✓	✓	✓		✓	✓		7
Ma et al., 2017		✓	✓	✓	✓	✓		✓	✓	✓	8
Liu J. W. et al., 2021		✓	✓	✓	✓		✓	✓	✓	✓	8
Liang et al., 2015		✓	✓	✓	✓	✓		✓	✓		7
Liu, 2019		✓	✓	✓	✓				✓		5
Wan et al., 2019		✓	✓	✓	✓	✓		✓	✓		7
Wu et al., 2022		✓	✓	✓	✓	✓		✓	✓	✓	8
Zhang et al., 2019b		✓	✓	✓	✓	✓		✓	✓		7
Fu et al., 2016a		✓	✓	✓	✓	✓		✓	✓		7
Wan et al., 2021			✓	✓	✓	✓			✓		5
Zeng et al., 2021		✓	✓	✓	✓	✓		✓	✓		7
Xiong et al., 2012		✓	✓	✓		✓		✓	✓	✓	7
Wang D. et al., 2021			✓	✓	✓				✓		4
Qin et al., 2022		✓	✓	✓	✓	✓		✓	✓		7
Zhou et al., 2018		✓	✓	✓	✓	✓	✓	✓	✓	✓	9
Zhu S. Q. et al., 2021		✓	✓	✓	✓	✓		✓	✓	✓	8

Q1, sample size calculation; Q2, statements describing control of temperature; Q3, randomly assigned to treatment or control groups; Q4, use of animals with Knee osteoarthritis; Q5, Evaluation of Knee osteoarthritis model building; Q6, use of anesthetic without marked intrinsic properties; Q7, blinded assessment of outcome; Q8, compliance with animal welfare regulations; Q9, published in a peer-reviewed journal or have passed peer review; and Q10, declared any potential conflict of interest.

### 3.3. Basic characteristics of the literature

Table 2 provides a summary of the features of the included studies. The animal models utilized in the included research were rabbit and rat, and the sex classification was pure male, pure female, half of each, and limitless sex, with a few studies not specified. The modeling methods included the Hulth-Telhag method, the Videman method, anterior cruciate ligament dissection, ovaries resection, femoral vein ligation, hind limb achilles tendon resection, external fixation of the hind knee, natural aging degeneration method, sodium iodoacetate solution injection, and LPS induction method. Acupuncture intervention methods include traditional acupuncture, electroacupuncture, acupuncture knife, and warm acupuncture.

**TABLE 2:** Study characteristics.

References	Species	Gender, amount	Model (method)	Treatment method	Course	Outcome index	Originate from
Chen et al., 2020	New Zealand rabbits	Un-limited, 8/group	Anterior cruciate ligament transection(ACLT)	Acupotomy	6 times	p38MAPK	Cartilaginous tissue of tibial plateau and femoral condyle
Fu et al., 2016b	New Zealand rabbits	Un-limited, 10/group	Hulth-Telhag	Needling	4 weeks	Caspase-3, Bax, Bcl-2, caspase-3 mRNA	Cartilaginous tissue of tibial plateau and femoral condyle
Liao et al., 2016	SD rat	Male, 10/group	Anterior cruciate ligament transection(ACLT)	Electroacupuncture	12 weeks	p38MAPK, mankin	Tibial plateau cartilage tissue
Zhu D. Y. et al., 2021	SD rat	Male,6/group	LPS inducing	Electroacupuncture	12 weeks	p38MAPK, P-p38MAPK, collagen II	Chondrocyte
Wang, 2020	Hartley guinea pigs	Female, 8/group	Spontaneity	Electroacupuncture	30 days	IL-1β	Tibial plateau cartilage tissue
Chen, 2021	SD rat	Male, 12/group	Induced by sodium iodoacetate solution	Electroacupuncture	4 weeks	IL-1β, MMP-13	Cartilaginous tissue of tibial plateau and femoral condyle
Liu D. et al., 2021	New Zealand rabbits	Un-limited,10/group	Cast fixation of knee joint in extension position	Warm Acupuncture	2 weeks	IL-1β, MMP-13, mankin	Cartilaginous tissue
Wu et al., 2019	New Zealand rabbits	Un-limited, 9/group	Hulth-Telhag	Electroacupuncture	8 weeks	IL-1β, mankin	Tibial plateau cartilage tissue
Wang et al., 2020	Hartley guinea pigs	Un-limited, 6/group	Spontaneity	Electroacupuncture	4 weeks	IL-1β, MMP-13	Cartilaginous tissue
Jie et al., 2021	SD rat	Male, 8/group	Anterior cruciate ligament transection (ACLT)	Electroacupuncture	12 weeks	MMP-13, ADAMTS-5, mankin	subchondral bone, Tibial plateau cartilage tissueand subchondral bone
Lin and Xu, 2019	Wistar rat	Male, 8/group	Modified videman	Needling	4 weeks	MMP-13	Cartilaginous tissue
Liu D. et al., 2017	New Zealand rabbits	Male, 10/group	Extension fixation of right hind limb	Warm Acupuncture	2 weeks	MMP-13, MMP-1	Tibial plateau cartilage tissue
Wu et al., 2018	New Zealand rabbits	Male, 10/group	Videman	Warm Acupuncture	2 weeks	MMP-13	Tibial plateau cartilage tissue
Xi et al., 2016	New Zealand rabbits	Un-limited, 10/group	Hulth-Telhag	Needling	4 weeks	MMP-13, collagen II	Meniscus and cartilage tissue
Zhang et al., 2019a	SD rat	Male, 10/group	Modified Hulth-Telhag	Electroacupuncture	12 weeks	MMP-13, mankin	Tibial plateau cartilage tissue
Bao et al., 2011	SD rat	Female, 10/group	Unilateral hindlimb Achilles tendinectomy	Needling	2 weeks	MMP-1	Cartilaginous tissue of the femoral condyle
Liu H. et al., 2021	rat	Male, 10/group	Anterior cruciate ligament transection (ACLT)	Needling	8 weeks	ADAMTS-5, collagen II, aggrecan, Apoptosis rate	Cartilaginous tissue
Liang, 2015	New Zealand rabbits	Male, 6/group	Modified extension cast fixation of knee joint	Acupotomy	3 times	Bax mRNA, Bcl-2 mRNA, Caspase-3 mRNA, Bax, Bcl-2, Caspase-3, mankin	Cartilaginous tissue of the femoral condyle
Yu, 2016	New Zealand rabbits	Male, 6/group	Modified Videman	Acupotomy	8 times	Collagen II, aggrecan, mankin	Cartilaginous tissue of tibial plateau and femoral condyle
Liu N. G. et al., 2018	New Zealand rabbits	Male, 6/group	Modified Videman	Acupotomy	4 weeks	Aggrecan	Cartilaginous tissue from the distal femur and proximal tibia
Ma et al., 2017	New Zealand rabbits	Male, 6/group	Videman	Electroacupuncture	4 weeks	Aggrecan	Cartilaginous tissue
Liu J. W. et al., 2021	rabbits	Half and half, 10/group	Extension fixation of right hind limb	Warm Acupuncture	2 weeks	Bax mRNA, Bcl-2 mRNA, Caspase-3 mRNA, Apoptosis rate, mankin	Cartilaginous tissue
Liang et al., 2015	New Zealand rabbits	Half and half, 6/group	Modified Videman	Acupotomy	3 times	Collagen II, aggrecan, mankin	Cartilaginous tissue of the femoral condyle
Liu, 2019	New Zealand rabbits	Half and half, 10/group	Extension fixation of right hind limb	Warm Acupuncture	2 weeks	Bax mRNA, Bcl-2 mRNA, Caspase-3 mRNA, Bax, Bcl-2, Caspase-3	Cartilaginous tissue of tibial plateau and femoral condyle
Wan et al., 2019	Wistar rat	Male, 8/group	Modified Videman	Needling	3 weeks	Caspase-3 mRNA, Caspase-3, Apoptosis rate	Cartilaginous tissue
Wu et al., 2022	New Zealand rabbits	Male, 10/group	Extension fixation of right hind limb	Warm Acupuncture	4 weeks	Bax mRNA, Bcl-2 mRNA, Bax, Bcl-2, Apoptosis rate, mankin	Articular cartilage of tibial plateau and subchondral bone
Zhang et al., 2019b	SD rat	Male, 10/group	Modified Hulth-Telhag	Electroacupuncture	12 weeks	Bax mRNA, Bcl-2 mRNA, Bax, Bcl-2, mankin	Tibial plateau cartilage tissue
Fu et al., 2016a	New Zealand rabbits	Un-limited, 10/group	Hulth-Telhag	Needling	4 weeks	p38MAPK, Apoptosis rate	Tibial plateau cartilage tissue
Wan et al., 2021	Wistar rat	Male, 8/group	Videman	Needling	3 weeks	Bax mRNA, Bcl-2 mRNA, Bax, Bcl-2	Cartilaginous tissue
Zeng et al., 2021	New Zealand rabbits	Male, 8/group	Modified Videman	Acupotomy	4 times	Caspase-3 mRNA, Caspase-3, mankin, Apoptosis rate	Cartilaginous tissue of the femoral condyle
Xiong et al., 2012	Japanese white rabbit	Male, 8/group	Ligate the femoral vein	Warm Acupuncture	8 weeks	Bax, Bcl-2, Apoptosis rate	Cartilaginous tissue of the femoral condyle
Wang D. et al., 2021	New Zealand rabbits	Male, 10/group	Extension fixation of right hind limb	Warm Acupuncture	4 weeks	Bax, Bcl-2, mankin	Tibial plateau cartilage tissue
Qin et al., 2022	New Zealand rabbits	Male, 6/group	Videman	Acupotomy	3 weeks	Mankin, BV/TV	Cartilage and subchondral bone
Zhou et al., 2018	SD rat	Female, 10/group	Ovariectomy	Electroacupuncture	12 weeks	Mankin, BV/TV, Tb.sp, Tb.Th, Tb.N	Cartilage and subchondral bone
Zhu S. Q. et al., 2021	New Zealand rabbits	Un-limited, 10/group	Hulth-Telhag	Needling	4 times	Tb.sp, Tb.Th, Tb.N	Tibial plateau cartilage tissue

### 3.4. Meta-analysis results

#### 3.4.1. p38MAPK pathway

**FIGURE 2:**
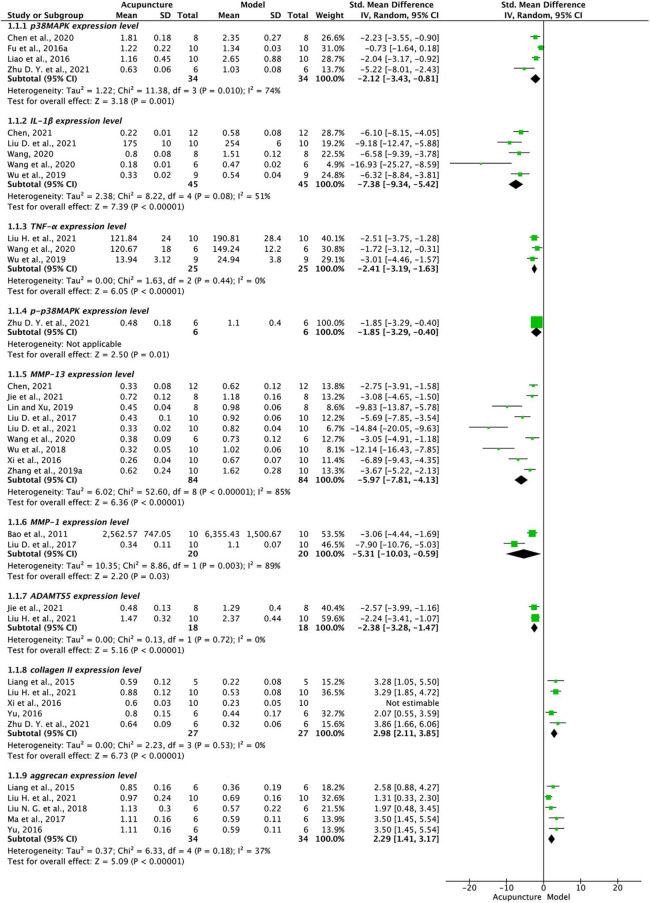
Forest plot of p38MAPK signaling regulators. p38MAPK, IL-1β, TNF-α, p-p38MAPK, MMP-13, MMP-1, ADAMST-5, collagen II, and aggrecan in acupuncture and model groups.

p38MAPK was utilized as an outcome measure in four investigations (Fu et al., 2016a; Liao et al., 2016; Chen et al., 2020; Zhu D. Y. et al., 2021). Acupuncture substantially lowered p38MAPK levels when compared to the control group (FEM, SMD –2.12, 95% CI: [–3.43,–0.81], P < 0.01; *X*^2^ = 11.38, *I*^2^ = 74%, Figure 2). At Revman, we ran a sensitivity analysis of p38MAPK by single exclusion and found that the effect size was steady and that removing one study had no influence on its significance. Similarly, five studies on IL-1β (Wu et al., 2019; Wang, 2020; Wang et al., 2020; Chen, 2021; Liu D. et al., 2021) and three on TNF-α (Wu et al., 2019; Wang et al., 2020; Liu H. et al., 2021) showed that all were significantly reduced in acupuncture intervention compared with model groups (*P* < 0.001, see Figure 2). One on p-p38MAPK (Zhu D. Y. et al., 2021) found significantly lower levels in acupuncture than in model groups (*P* = 0.01, Figure 2).

MMP-13 was used as an outcome indicator in nine studies (Xi et al., 2016; Liu D. et al., 2017; Wu et al., 2018; Lin and Xu, 2019; Zhang et al., 2019a; Wang et al., 2020; Chen, 2021; Jie et al., 2021; Liu D. et al., 2021), and pooled analysis showed that acupuncture significantly reduced MMP-13 compared to model groups (*P* < 0.001, *I*^2^ = 85%, Figure 2). The single exclusion method showed a stable effect. In two studies on MMP-1 (Bao et al., 2011; Liu D. et al., 2017) and two on ADAMTS5 (Jie et al., 2021; Liu H. et al., 2021) levels were also significantly lower in acupuncture treatment than in model groups, their results were *P* = 0.03 and *P* < 0.001, respectively, see Figure 2. Conversely, five studies on collagen II (Liang et al., 2015; Xi et al., 2016; Yu, 2016; Liu H. et al., 2021; Zhu D. Y. et al., 2021) and five on aggrecan (Liang et al., 2015; Yu, 2016; Ma et al., 2017; Liu N. G. et al., 2018; Liu H. et al., 2021), found significantly higher levels in acupuncture than in model groups (*P* < 0.001, Figure 2), heterogeneity of collagen II was significantly lower after exclusion of one study (Xi et al., 2016) perhaps due to sex distribution differences between studies(unrestricted in that study, all male or half male in others).

#### 3.4.2. Mitochondrial pathway

##### 3.4.2.1. mRNA expression of cytokines on the mitochondrial signaling pathway in cartilage

**FIGURE 3:**
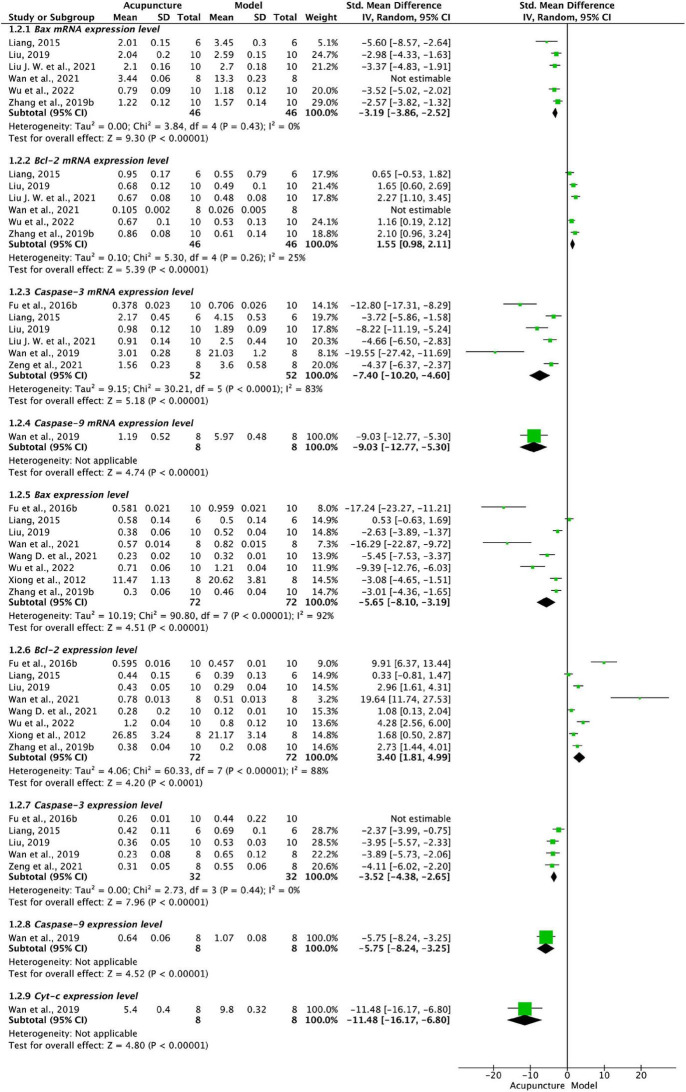
Forest plot of signaling regulator of mitochondria pathway. Bax mRNA, Bcl-2 mRNA, Caspase-3 mRNA, Caspase-9 mRNA, Bax, Bcl-2, Caspase-3, Caspase-9, Cyt-c in acupuncture and model groups.

As shown by Figure 3, six publications (Liang, 2015; Liu, 2019; Zhang et al., 2019b; Liu J. W. et al., 2021; Wan et al., 2021; Wu et al., 2022) showed that Bax mRNA was significantly reduced in acupuncture compared to model groups (*P* < 0.001). However, in the same studies Bcl-2 mRNA was elevated in acupuncture groups compared to model groups. Sensitivity analysis showed that the heterogeneities of both Bax mRNA and Bcl-2 mRNA were significantly lower after excluding one study (Wan et al., 2021). The measured value in this study appeared small compared with other studies and this may be due to high RNA degradation rate or the concentration of internal reference genes of the samples sent for testing.

Six studies (Liang, 2015; Fu et al., 2016b; Liu, 2019; Wan et al., 2019; Liu J. W. et al., 2021; Zeng et al., 2021) on Caspase-3 mRNA and one (Wan et al., 2019) on Caspase-9 mRNA were significantly lower in acupuncture than model groups (*P* < 0.001, Figure 3). Single exclusion method shows stable results in Caspase 3.

##### 3.4.2.2. Protein expression of cytokines on the mitochondrial signaling pathway in cartilage

Figure 3 shows that pooled data from eight studies (Xiong et al., 2012; Liang, 2015; Fu et al., 2016b; Liu, 2019; Zhang et al., 2019b; Wan et al., 2021; Wang D. et al., 2021; Wu et al., 2022) found reduced Bax and increased Bcl-2 in acupuncture compared to model groups (*P* < 0.001). The single exclusion method showed a stable effect.

Pooled analysis from five studies (Liang, 2015; Fu et al., 2016b; Liu, 2019; Wan et al., 2019; Zeng et al., 2021) shows that Caspase-3 is significantly reduced in acupuncture compared to model groups (*P* < 0.001, Figure 3). Sensitivity analysis showed a significant reduction in heterogeneity after excluding one study (Fu et al., 2016b). This may be due to the unrestricted sex distribution of the animals used in that study, while other studies were all-male (Liang, 2015; Wan et al., 2019; Zeng et al., 2021) or half-male (Liu, 2019).

One study (Wan et al., 2019) found reduced Caspase-9 and Cytochrome-c (Cyt-c) levels in acupuncture compared with a model group (*P* < 0.001, Figure 3).

#### 3.4.3. Effect of acupuncture intervention on cartilage phenotype in KOA

##### 3.4.3.1. Chondrocyte apoptosis rate

**FIGURE 4:**
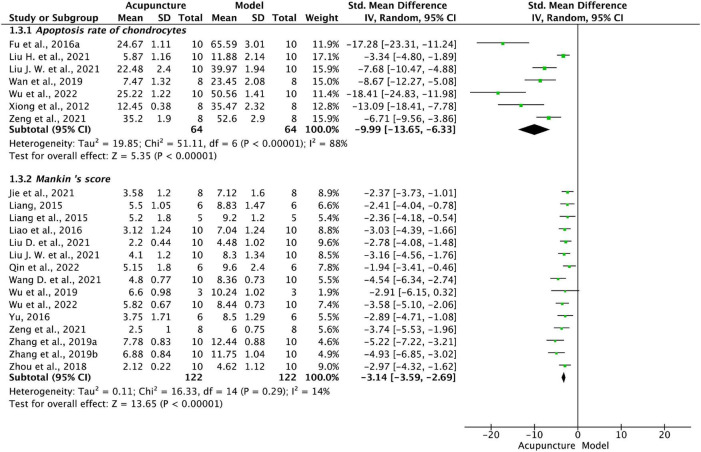
Forest plot of apoptosis rate and Mankin’s score.

Pooled data from seven studies (Xiong et al., 2012; Fu et al., 2016a; Wan et al., 2019; Liu H. et al., 2021; Liu J. W. et al., 2021; Zeng et al., 2021; Wu et al., 2022) showed that chondrocyte apoptosis rate was significantly lower in acupuncture than model groups (*P* < 0.001, *I*^2^ = 88%, Figure 4). The single exclusion method showed a stable effect.

##### 3.4.3.2. Mankin’s score

Cartilage damage is the gold standard for KOA assessment, and the Mankin’s score as revised by the Osteoarthritis Research Society International (OARSI) (Moskowitz, 2006) is the most commonly used system to gauge cartilage destruction. Higher scores indicate more severe cartilage breakdown, lower chondrocyte counts, and worse pathological staining. As shown by Figure 4, data from 15 studies (Liang, 2015; Liang et al., 2015; Liao et al., 2016; Yu, 2016; Zhou et al., 2018; Wu et al., 2019, 2022; Zhang et al., 2019a,b; Jie et al., 2021; Liu D. et al., 2021; Liu J. W. et al., 2021; Wang D. et al., 2021; Zeng et al., 2021; Qin et al., 2022) show significantly lower score in acupuncture than model groups (*P* < 0.001).

##### 3.4.3.3. Cartilage morphometrics

**FIGURE 5:**
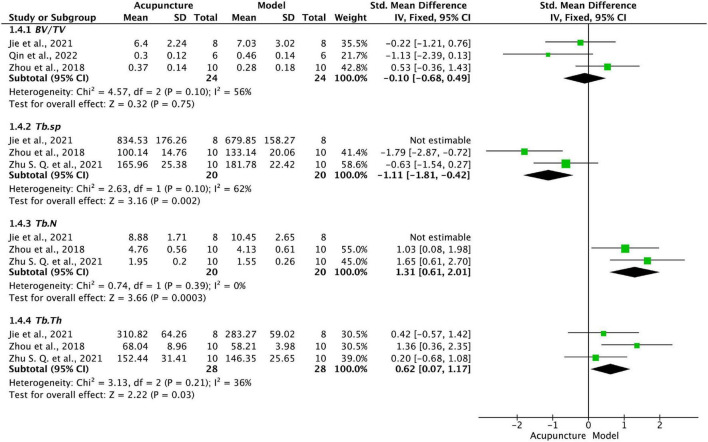
Forest plot of morphometry of cartilage.

To assess the extrinsic effects of acupuncture on the regulation of matrix-degrading enzymes and extracellular matrix molecules in cartilage, we included the following values that quantify cartilage morphometry:

Data from three studies (Zhou et al., 2018; Jie et al., 2021; Qin et al., 2022) showed that BV/TV scores did not differ significantly between the acupuncture and model groups (*P* = 0.75, Figure 5). Further pooled data from three studies (Zhou et al., 2018; Jie et al., 2021; Zhu S. Q. et al., 2021) showed significant reduction in Tb.sp scores (*P* < 0.01, Figure 5) and significant increase in both Tb,N (*P* < 0.001, Figure 5)and Tb.Th (*P* < 0.05, Figure 5) in acupuncture compared with model groups. Sensitivity analysis showed a significant decrease in Tb.sp and Tb.N heterogeneity after excluding one study (Jie et al., 2021). We found that the values of the outcome indicators varied considerably among studies, probably due to the different magnifications of the light microscope and the software used for the calculation.

### 3.5. Subgroup analysis

Sources of a high level of heterogeneity (*I*^2^ = 85%) among studies on MMP-13 were explored from two subgroup analyses differing according to clinical characteristics, as follows.

**FIGURE 6:**
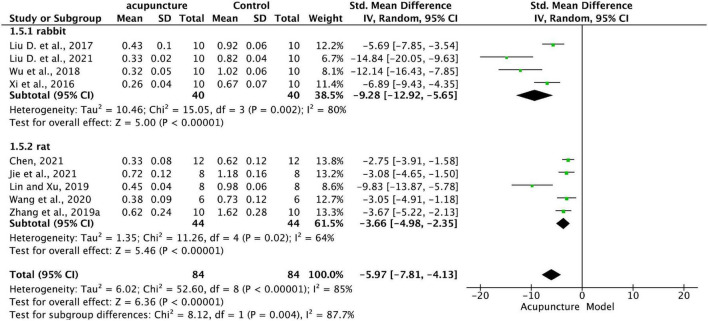
Subgroup analysis of effects of acupuncture on MMP-13 in KOA cartilage in different animal models (rabbit and rat).

**FIGURE 7:**
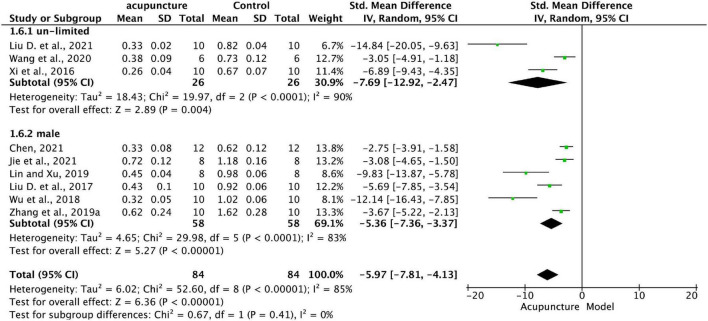
Subgroup analysis of MMP-13 in KOA cartilage treated with acupuncture in animal models of different gender distributions (male and un-limited).

(1) Articles were allocated to subgroups according to animal species (rabbit and rat). Results of analysis presented in Figure 6 show that the expression of MMP-13 in the acupuncture group was all significantly lower than that in the model group (*P* < 0.001), species factors don’t influence acupuncture to reduce MMP-13 expression. However, the combined forest plot effect shows a more pronounced reduction in the leftward shift of the effector amount of MMP-13 in the cartilage of rabbits compared to rats. Furthermore, the difference between subgroups reached statistical significance (*P* = 0.004), with a greater reduction in the rabbit subgroup. These results suggest that the magnitude of the efficacy of acupuncture may be species dependent and that rabbits may be relatively more sensitive to the effects of acupuncture.

(2) Study data were allocated to subgroups according to sex selection (sex-unlimited and male), and the results of the analysis in Figure 7 show that the effect on MMP-13 was significantly greater in acupuncture than model groups in both forms of sex selection, providing further evidence of the generalizability of acupuncture for KOA. The decline of MMP-13 in male (*P* < 0.001) is more prominent than in sex-unlimited (*P* = 0.004), however, the differences between subgroups didn’t reach statistical significance (*P* = 0.41).

### 3.6. Sensitivity analysis

**FIGURE 8:**
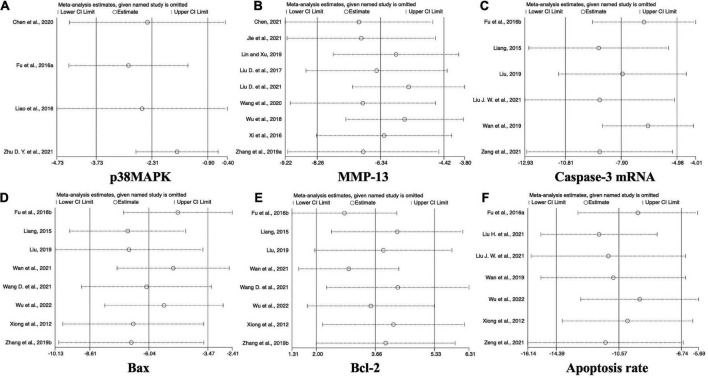
Sensitivity analysis of acupuncture effects on p38MAPK, MMP-13, Caspase-3 mRNA, Bax, and Bcl-2 in KOA cartilage.

To evaluate the stability of the results of acupuncture studies on KOA animal models, studies with significant differences but high heterogeneity (containing p38MAPK, MMP-13, Caspase-3 mRNA, Bax, Bcl-2 expression levels, and chondrocyte apoptosis rates) were subjected to sensitivity analysis using Stata 17.0 to explore the sources of heterogeneity and the degree of influence on the combined effect sizes. The results of this analysis showed that data from all studies were evenly distributed around the line of no difference, and the MD, confidence interval, and heterogeneity did not change significantly with each excluded study, indicating that the results were relatively robust (Figure 8).

### 3.7. Publication bias

**FIGURE 9:**
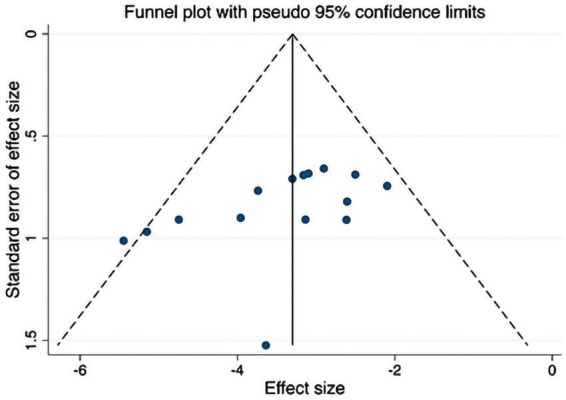
Funnel plots of Mankin’s score in the KOA animal model of acupuncture intervention.

The funnel plot and the Egger test were used to compensate for publication bias; if *P* < 0.05 in the both Egger’s test and Begg’s test indicate potential bias. In this study, funnel plots for the effect of acupuncture on the Mankin’s score of cartilage were created. The Egger’s test, *P* = 0.051, close to 0.05, combined with the results of Begg’s test (*P* = 0.018) and the asymmetry of the funnel plot, prefers to consider a slight publication bias, indicating a possible bias. This is depicted in Figure 9.

## 4. Discussion

### 4.1. Summary of main findings

In this review, we systematically analyzed 40 acupuncture studies on cytokines relevant to p38MAPK and mitochondrial pathways in cartilage samples from animal models. We found that in cartilage, acupuncture significantly inhibited two important KOA-inducing mediators, IL-1β and TNF-α, attenuating their effects on the cartilage environment. It also significantly blocked their activation of p38MAPK, preventing activation of the MAPK signaling pathway in cartilage and allowing increased P38MAPK phosphorylation (p-p38MAPK) (Park et al., 2018). Acupuncture also significantly attenuates IL-1β and TNF-α activation of MAPK and inhibits the expression of p-p38 MAPK. Reduced p-p38MAPK limits the production of enzymes such as MMP and ADAMTS that degrade and cause phenotypic change in cartilage matrix (Wang et al., 2013; Lieberthal et al., 2015). MMP-13, the main MMP involved in cartilage degradation (Mehana et al., 2019), has the specific ability to cleave collagen II and degrade aggrecan molecules, playing a dual role in matrix destruction, which makes it an attractive target for the treatment of OA. The ADAMTS protein family has also been associated with cartilage degradation of KOA, particularly ADAMTS5 (Kapoor et al., 2011). Therefore, MMP13 and ADAMTS5 were used as catabolic markers, while collagen II and aggrecan were used as anabolic markers of cartilage metabolism (He et al., 2020). The regulated synthesis or activity of these enzymes is essential to inhibit cartilage degeneration and catabolism of the cartilage matrix, and we found that acupuncture intervention significantly inhibited the expression of MMP-13 and ADAMTS5 thereby significantly reversing the reduction of collagen and aggrecan proteins in the extracellular matrix of cartilage during KOA progression.

In addition, the catabolism of cartilage matrix in KOA leads to chondrocyte apoptosis and a decrease in chondrocyte survival signals (Sun et al., 2019). Bcl-2 and Bax protein levels are directly related to apoptosis. Elevated Bcl-2 can inhibit apoptosis by moderating mitochondrial permeability. Conversely, elevated Bax can promote apoptosis by increasing mitochondrial membrane permeability via activated oligomers and Cyt-C release into the cytoplasm. The latter activates key enzymes of the mitochondria-dependent apoptotic pathway, triggering the Caspase cascade, with sequential activation of Caspase-9 and Caspase-3, leading to apoptosis (Vakifahmetoglu-Norberg et al., 2017). In the present study, acupuncture significantly modulated the mRNA and protein expression levels of Bcl-2, Bax, Caspase-9, and Caspase-3, inhibited the expression of Cyt-c, and reduced the chondrocyte apoptosis rate, suggesting that acupuncture achieved this by inhibiting the activation of mitochondrial pathways.

We also reviewed relevant phenotypes of cartilage to verify the effectiveness of the acupuncture effect. To verify cartilage improvement, chondrocyte apoptosis rates from Tunnel or cell flow tests, the Mankin score (which uses electron microscopy and pathological staining to assess cartilage gross morphological structure), and indicators of morphometric changes in cartilage and subchondral bone observed using light microscopy (BV/TV, Tb.sp, Tb.N, Tb.Th) were included. The results showed that acupuncture significantly improved the apoptosis of chondrocytes and the repair of cartilage morphological structures, especially the mean thickness of trabecular column structures (Tb.Th), the mean number of trabeculae per unit length (Tb.N), and the mean distance between trabeculae (column structures; Tb.Sp), providing strong evidence for cartilage remodeling. The exceptions were cartilage and BV/TV, which did not differ significantly, a surprising finding which may be due to the small number of included studies and the range of modeling methods, and needs to be verified by further study.

Species factors have significant effects on the mechanistic study of acupuncture. In the subgroup analysis of species differences, we found that acupuncture was effective across species, providing further evidence for the generalizability of acupuncture for KOA. The relevant forest plot (Figure 9) showed that acupuncture may be relatively sensitive in rabbits, consistent with a study (Vina et al., 2021) which found treatment differences in acupuncture management of KOA between African American and white participants, possibly due to cognitive selection bias by race or differences in response to mind-body feedback.

Furthermore, acupuncture performed effectively for persons with KOA regardless of gender, which is consistent with a secondary analysis of a multicenter randomized controlled study revealing that the efficacy of acupuncture for KOA is independent of gender (Hao et al., 2022). While we discovered in the forest plot that acupuncture may work more sensitively in men, some studies have examined gender differences in the efficacy of acupuncture management for KOA and the cost-effectiveness of treatment (Reinhold et al., 2008; Vina et al., 2021), indicating that acupuncture is more effective in women with osteoarthritis and that women are more favorable to acupuncture, the underlying reasons for this gender difference remain unknown. The fact that acupuncture engages distinct active brain regions in men and women may explain the gender disparities in acupuncture benefits (Li et al., 2018).Thus, demographics and gender demand additional investigation and emphasize the necessity for thorough control of these factors in mechanistic research.

### 4.2. Strengths and limitations

To our knowledge, this is the first systematic review of the effects of acupuncture on KOA pathway mechanisms and related phenotypes in animal studies and the first study to evaluate acupuncture interventions in KOA animal experiments, providing a reference for future KOA mechanism studies and animal experiments. We conducted as comprehensive a search as possible for the full text of all articles (in any language) in nine databases. The CAMARADES inventory was used to assess the quality of the studies and to extract data for analysis to validate our findings. The present study was conducted to provide a theoretical basis for revealing the mechanism of acupuncture action in the future.

However, there are some limitations to this research. The studies we included did not perform p38MAPK and mitochondrial pathway silencing experiments, which should be conducted in future studies on acupuncture mechanisms. Secondly, acupuncture is a traditional Chinese therapy, and the included literature is from China, which may have an unavoidable publication bias. Moreover, the small amount and poor quality of the selected literature meant that no firm conclusions could be drawn. We look forward to higher quality studies on the mechanisms of acupuncture and hope that the present review provides a useful reference in the field.

## 5. Conclusion

In conclusion, our analysis shows that acupuncture treatments might minimize cartilage extracellular matrix breakdown by inhibiting cytokine production in the p38MAPK pathway and regulating cytokines in the mitochondrial route to limit chondrocyte death. It also appears to enhance cartilage tissue morphologies, preventing cartilage degradation.

## Data availability statement

The original contributions presented in this study are included in the article/Supplementary material, further inquiries can be directed to the corresponding author.

## Author contributions

J-NY took responsibility for the integrity of the data and the accuracy of the data analysis. J-NY and C-GS drafted the manuscript and performed statistical analysis. Y-QJ, YZ, W-XS, X-XZ, J-TM, and X-YL made critical revision of the manuscript for important intellectual content. JZ supervised the study. All authors conceptualized, designed the study, analyzed, and interpreted data.

## Footnotes

1
https://inplasy.com/inplasy-2022-9-0125/
