# Supplementary material for: Effects of acupuncture on cartilage p38MAPK and mitochondrial pathways in animal model of knee osteoarthritis: A systematic evaluation and meta-analysis
Source: Front Neurosci. 2023 Jan 11;16:1098311. doi: 10.3389/fnins.2022.1098311 (PMC9875597; doi:10.3389/fnins.2022.1098311)
Supplement: Supplementary file 1 [file Data_Sheet_1.PDF]

Supplementary Material

Search strategy

1.Search strategy used in PubMed database.

#1. Animal Model [MeSH Terms] OR rat [Title/Abstract] OR rats [Title/Abstract] OR mouse [Title/Abstract] OR mice [Title/Abstract] OR Rabbit [Title/Abstract] OR Rabbits [Title/Abstract] OR dog [Title/Abstract] OR pig [Title/Abstract] OR animal [Title/Abstract] OR animals [Title/Abstract]

Items found: 3963796

#2. Osteoarthritis, Knee [MeSH Terms] OR Knee Osteoarthritides [Title/Abstract] OR Knee Osteoarthritis [Title/Abstract] OR Osteoarthritis of Knee [Title/Abstract] OR Osteoarthritis of the Knee [Title/Abstract] Items found: 30699

#3. acupuncture[MeSH Terms] OR acupun* [Title/Abstract] OR needl* therapy [Title/Abstract] OR needl* treat* [Title/Abstract] OR moxibustion [Title/Abstract] OR acupuncture with electrical stimulation [Title/Abstract] OR electro-acupuncture [Title/Abstract] OR electric acupuncture [Title/Abstract] OR laser acupuncture [Title/Abstract] OR photo-acupuncture [Title/Abstract] OR auricular [Title/Abstract] OR ear acupuncture [Title/Abstract] OR face acupuncture [Title/Abstract] OR hand acupuncture [Title/Abstract] OR scalp acupuncture [Title/Abstract] OR acupressure [Title/Abstract] OR Pharmacoacupuncture [Title/Abstract] Items found: 94292

#4. #1 AND #2 AND #3 Items found: 62

2. Search strategy used in Web of Science database

#1. TS=(acupuncture) OR AB=(acupun* OR needl* therapy OR needl* treat* OR moxibustion OR acupuncture with electrical stimulation OR electro-acupuncture OR electric acupuncture OR laser acupuncture OR photo- acupuncture OR auricular OR ear acupuncture OR face acupuncture OR hand acupuncture OR scalp acupuncture OR acupressure OR Pharmacoacupuncture) Items found: 42310

#2. TS=(Osteoarthritis, Knee) OR AB=(Knee Osteoarthritides OR Knee Osteoarthritis OR Osteoarthritis of Knee OR Osteoarthritis of the Knee) Items found: 45168

#3. TS=(Animal Model) OR AB=(rat OR rats OR mouse OR mice OR Rabbit OR Rabbits OR dog OR pig OR animal OR animals) Items found:2847670

#4. #1 AND #2 AND #3 Items found: 78

3. Search strategy used in cochrane library

#1. MeSH descriptor: [Acupuncture] explode all trees Items found: 164

#2. (Acupuncture or acupun* or needl* therapy or needl* treat* or moxibustion or acupuncture with electrical stimulation or electro-acupuncture or electric acupuncture or laser acupuncture or photo-acupuncture or auricular or ear acupuncture or face acupuncture or hand acupuncture or scalp acupuncture or acupressure or Pharmacoacupuncture):ti,ab,kw Items found: 28682

#3.#1 or #2 Items found: 28682

#4. MeSH descriptor: [Osteoarthritis, Knee] explode all trees Items found:5202

#5. (Osteoarthritis, Knee or Knee Osteoarthritides or Knee Osteoarthritis or Osteoarthritis of Knee or Osteoarthritis of the Knee):ti,ab,kw Items found:14815

#6.#4 or #5 Items found:14815

#7. MeSH descriptor: [Models, Animal] explode all trees Items found:531

#8. (Models, Animal or rat or rats or mouse or mice or Rabbit or Rabbits or dog or pig or animal or animals):ti,ab,kw Items found:39764

#9.#7 or #8 Items found:39772

#10.#3 and #6 and #9 Items found:12

4. Search strategy used in Scopus

#1. TITLE-ABS-KEY (“Models, Animal” OR “Models, Animal” OR rat OR rats OR mouse OR mice OR Rabbit OR Rabbits OR dog OR pig OR animal OR animals) Items found: 8659938

#2. TITLE-ABS-KEY (Acupuncture OR acupun* OR needl* therapy OR needl* treat* OR moxibustion OR “acupuncture with electrical stimulation” OR electro-acupuncture OR “electric acupuncture” OR “laser acupuncture” OR photo-acupuncture OR auricular OR “ear acupuncture” OR “face acupuncture” OR “hand acupuncture” OR “scalp acupuncture” OR acupressure OR Pharmacoacupuncture) Items found: 102803

#3. TITLE-ABS-KEY (“Osteoarthritis, Knee” OR “Knee Osteoarthritides” OR “Knee Osteoarthritis” OR “Osteoarthritis of Knee” OR “Osteoarthritis of the Knee”) Items found: 42250

#4. #1 AND #2 AND #3 Items found:62

5. Search strategy used in CNKI

SU=('膝骨关节炎'+'骨关节炎'+'膝痹'+'膝骨关节病'+'膝关节骨关节炎'+'膝骨性关节炎'+'膝关节骨性关节炎')*('针灸'+'针刺'+'艾灸'+'物理疗法'+'电针'+'三棱针'+'耳针'+'体针'+'腕踝针'+'刺灸'+'

刺法'+'新针疗法'+'电热针'+'管针'+'毫针'+'温针'+'火针'+'激光针'+'头针'+'穴位注射'+'埋线'+'埋针'+'皮内针'+'穴位'+'耳穴'+'经络'+'刺络')*('动物模型'+'动物实验'+'实验'+'动物'+'小鼠'+'犬'+'大鼠'+'兔'+'猪') 检索结果：425 中文

6. Search strategy used in Wan Fang

(主题:(膝骨关节炎) or 题名或关键词:(骨关节炎 or 膝痹 or 膝骨关节病 or 膝关节骨关节炎 or 膝骨性关节炎 or 膝关节骨性关节炎)) and (主题:(针灸) or 题名或关键词: (针刺 or 艾灸 or 物理疗法 or 电针 or 三棱针 or 耳针 or 体针 or 腕踝针 or 刺灸 or 刺法 or 新针疗法 or 电热针 or 管针 or 毫针 or 温针 or 火针 or 激光针 or 头针 or 穴位注射 or 埋线 or 埋针 or 皮内针 or 穴位 or 耳穴 or 经络 or 刺络)) and (主题:(动物模型) or 题名或关键词:(动物实验 or 实验 or 动物 or 小鼠 or 犬 or 大鼠 or 兔 or 猪)) 检索结果：547

7. Search strategy used in VIP

M=(膝骨关节炎 OR 骨关节炎 OR 膝痹 OR 膝骨关节病 OR 膝关节骨关节炎 OR 膝骨性关节炎 OR 膝关节骨性关节炎)AND M=(针灸 OR 针刺 OR 艾灸 OR 物理疗法 OR 电针 OR 三棱针 OR 耳针 OR 体针 OR 腕踝针 OR 刺灸 OR 刺法 OR 新针疗法 OR 电热针 OR 管针 OR 毫针 OR 温针 OR 火针 OR 激光针 OR 头针 OR 穴位注射 OR 埋线 OR 埋针 OR 皮内针 OR 穴位 OR 耳穴 OR 经络 OR 刺络)AND M=(动物模型 OR 动物实验 OR 实验 OR 动物 OR 小鼠 OR 犬 OR 大鼠 OR 兔 OR 猪) 检索结果：214

8. Search strategy used in Embase

#1.acupuncture:ab,ti OR acupun*:ab,ti OR 'needl* therapy':ab,ti OR 'needl* treat*':ab,ti OR moxibustion:ab,ti OR 'acupuncture with electrical stimulation':ab,ti OR 'electro acupuncture':ab,ti OR 'electric acupuncture':ab,ti OR 'laser acupuncture':ab,ti OR 'photo-acupuncture':ab,ti OR auricular:ab,ti OR 'ear acupuncture':ab,ti OR 'face acupuncture':ab,ti OR 'hand acupuncture':ab,ti OR 'scalp acupuncture':ab,ti OR acupressure:ab,ti OR pharmacoacupuncture:ab,ti Items found:52801

#2.'osteoarthritis, knee':ab,ti OR 'knee osteoarthritides':ab,ti OR 'knee osteoarthritis':ab,ti OR 'osteoarthritis of knee':ab,ti OR 'osteoarthritis of the knee':ab,ti Items found:26033

#3.'animal model':ab,ti OR rat:ab,ti OR rats:ab,ti OR mouse:ab,ti OR mice:ab,ti OR rabbit:ab,ti OR rabbits:ab,ti OR dog:ab,ti OR pig:ab,ti OR animal:ab,ti OR animals:ab,ti Items found:4657592

#1 AND #2 AND#3 Items found:56

9. Search strategy used in MedLINE

#1. exp Acupuncture Points/ or exp Acupuncture, Ear/ or exp Acupuncture/ or exp Acupuncture Therapy/ or exp Electroacupuncture/ or exp electric acupuncture/ or exp Electric Stimulation Therapy/ or exp Acupressure/ or exp face acupuncture/ or exp hand acupuncture/ or exp photo-acupuncture / or exp scalp acupuncture / or exp moxibustion/ or exp laser acupuncture/ or exp hand acupuncture/ Items found:113903

#2. (acupuncture$ or "fire needling" or "warm needling" or "pyonex" or "intradermal needling" or "pharmacoacupuncture").ab,ti. Items found:25436

#3. 1 or 2 Items found:120055

#4. Osteoarthritis, Knee/ Items found:25410

#5. Osteoarthritis.mp Items found:104882

#6. exp osteoarthritis, knee/ or exp osteoarthritis/ Items found:73762

#7. ("osteoarthritis, knee" or "knee osteoarthritides" or "knee osteoarthritis" or "osteoarthritis of knee" or "osteoarthritis of the knee").ab,ti Items found:17422

#8. 4 or 5 or 6 or 7 Items found:104882

#9. exp animals/ Items found:25760263

#10. exp Animal Model/ or exp rat/ or exp rats/ or exp mouse/ or exp mice/ or exp Rabbit/ or exp Rabbits/ or exp dog/ or exp pig/ or exp animal/ Items found: 25761916

#11. 9 or 10 Items found: 25761916

#12. exp humans/ not animals.sh. Items found:18564933

#13. 11 not 12 Items found: 7196983

#14. 13 and 8 and 3 Items found:104
